# Diaqua­bis­(1,10-phenanthroline-κ^2^ *N*,*N*′)manganese(II) sulfate hexa­hydrate

**DOI:** 10.1107/S160053681004211X

**Published:** 2010-10-23

**Authors:** Chun Zhang, Hong-lin Zhu

**Affiliations:** a State Key Laboratory Base of Novel Functional Materials and Preparation Science, Center of Applied Solid State Chemistry Research, Ningbo University, Ningbo, Zhejiang 315211, People’s Republic of China

## Abstract

In the title compound, [Mn(C_12_H_8_N_2_)_2_(H_2_O)_2_]SO_4_·6H_2_O, the complex cations assemble into positively charged sheets parallel to (010) *via* inter­molecular π–π stacking inter­actions with a mean interplanar distance of 3.410 (6) along [100] and 3.465 (5) Å along [001]. The sulfate anions and uncoordinated water mol­ecules are inter­connected between these layers by hydrogen bonds, forming negatively charged layers which are linked to the positive layers through O—H⋯O hydrogen bonds, forming a three-dimensional architecture. Both the positive and negative sheets are stacked along [010] in an ⋯*ABAB*⋯ sequence, the *A* layers being shifted by 1/2*a* along [100] with respect to the *B* layers. One of the uncoordinated water molecules is equally disordered over two positions.

## Related literature

For general background, see: Sangeetha & Maitra (2005 ▶); Lehn (2007 ▶); Stang & Olenyuk (1997 ▶). For related structures, see: Devereux *et al.* (2000 ▶); Zheng *et al.* (2003 ▶); Zhang *et al.* (2003 ▶, 2005 ▶).
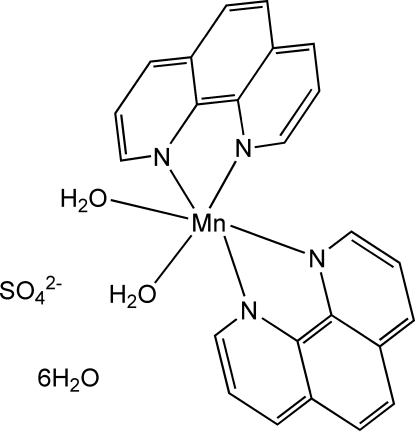


## Experimental

### 

#### Crystal data

[Mn(C_12_H_8_N_2_)_2_(H_2_O)_2_]SO_4_·6H_2_O
*M* *_r_* = 655.54
Triclinic, 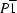

*a* = 10.153 (2) Å
*b* = 12.086 (2) Å
*c* = 13.309 (3) Å
α = 109.55 (3)°
β = 91.79 (3)°
γ = 110.56 (3)°
*V* = 1420.2 (5) Å^3^
*Z* = 2
Mo *K*α radiation
μ = 0.61 mm^−1^
*T* = 293 K
0.29 × 0.24 × 0.19 mm

#### Data collection

Rigaku R-AXIS RAPID diffractometer
Absorption correction: multi-scan (*ABSCOR*; Higashi, 1995 ▶) *T*_min_ = 0.680, *T*_max_ = 0.843
13888 measured reflections
6388 independent reflections
5780 reflections with *I* > 2σ(*I*)
*R*_int_ = 0.022

#### Refinement

*R*[*F* ^2^ > 2σ(*F* ^2^)] = 0.048
*wR*(*F* ^2^) = 0.147
*S* = 1.19
6388 reflections
382 parameters
H-atom parameters constrained
Δρ_max_ = 0.56 e Å^−3^
Δρ_min_ = −0.58 e Å^−3^

### 

Data collection: *RAPID-AUTO* (Rigaku, 1998 ▶); cell refinement: *RAPID-AUTO*; data reduction: *CrystalStructure* (Rigaku/MSC, 2004 ▶); program(s) used to solve structure: *SHELXS97* (Sheldrick, 2008 ▶); program(s) used to refine structure: *SHELXL97* (Sheldrick, 2008 ▶); molecular graphics: *ORTEPII* (Johnson, 1976 ▶); software used to prepare material for publication: *SHELXL97*.

## Supplementary Material

Crystal structure: contains datablocks global, I. DOI: 10.1107/S160053681004211X/gw2091sup1.cif

Structure factors: contains datablocks I. DOI: 10.1107/S160053681004211X/gw2091Isup2.hkl

Additional supplementary materials:  crystallographic information; 3D view; checkCIF report

## Figures and Tables

**Table 1:** Hydrogen-bond geometry (Å, °)

*D*—H⋯*A*	*D*—H	H⋯*A*	*D*⋯*A*	*D*—H⋯*A*
O1—H1*B*⋯O5	0.86	1.82	2.670 (4)	174
O1—H1*C*⋯O7	0.86	1.99	2.843 (3)	178
O2—H2*B*⋯O3	0.85	1.83	2.656 (3)	164
O2—H2*C*⋯O3^i^	0.86	1.84	2.684 (3)	168
O7—H7*A*⋯O8^ii^	0.86	2.00	2.856 (3)	176
O7—H7*B*⋯O10^ii^	0.86	1.98	2.799 (4)	160
O8—H8*B*⋯O6	0.86	2.01	2.842 (4)	165
O8—H8*C*⋯O11^iii^	0.86	1.93	2.778 (4)	171
O9—H9*B*⋯O5	0.85	1.85	2.704 (4)	174
O9—H9*C*⋯O12*A*	0.86	1.98	2.617 (7)	131
O10—H10*B*⋯O9^iv^	0.85	2.04	2.836 (5)	157
O10—H10*C*⋯O9^ii^	0.86	2.02	2.875 (5)	172
O11—H11*A*⋯O12*B*^v^	0.77	1.93	2.691 (7)	166
O11—H11*B*⋯O6	0.85	1.98	2.813 (4)	167
O12*A*—H12*A*⋯O4	1.15	1.87	2.827 (6)	138
O12*B*—H12*B*⋯O4^v^	0.86	2.01	2.851 (6)	164

Symmetry codes: (i) 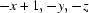; (ii) 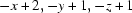; (iii) 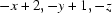; (iv) 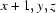; (v) 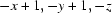.

**Fig. 1.**
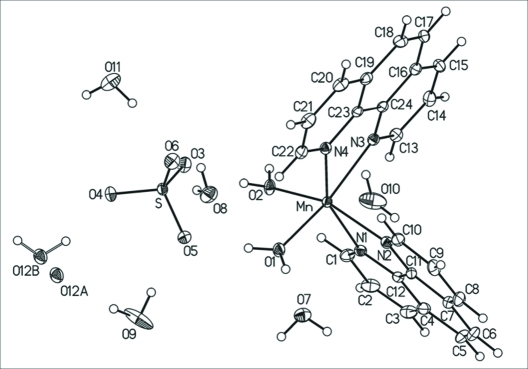
ORTEP view of the title compound. The dispalcement ellipsoids are drawn at 45% probability level.

**Fig. 2.**
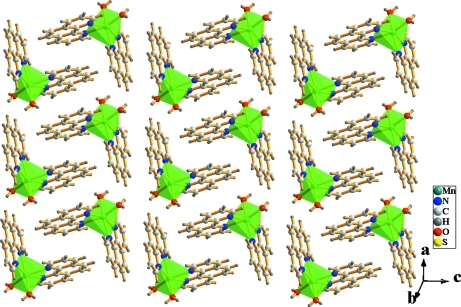
The positively charged two-dimensional layer of the complex cations pallel to (010).

**Fig. 3.**
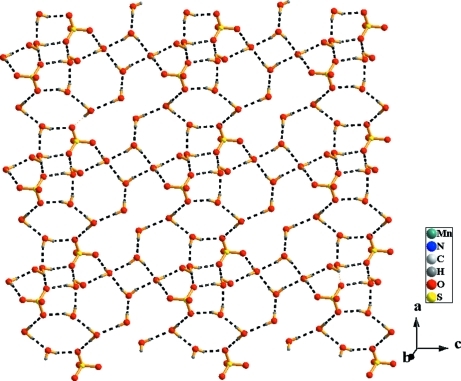
The negatively charged two-dimensional layer of the sulfate anions and crystal water molecules pallel to (010).

**Fig. 4.**
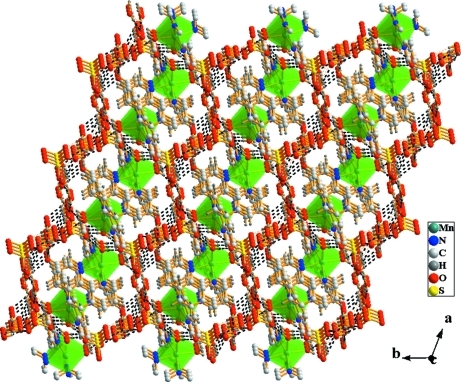
The three-dimensional structure of the title compound.

## supplementary crystallographic information

### Comment

Construction of supramolecular architectures with interesting physical properties have been one of the most active fields in supramolecular chemistry, coordination chemistry and materials science owing to their potential use as new functional materials (Sangeetha *et al.*, 2005; Lehn, 2007). The most efficient and widely used approach for designing such materials is the self-assembly of organic ligands and metal ions (Stang *et al.*, 1997). Here, we report a Mn(II) complex {[Mn(H_2_O)_2_(C_12_H_8_N_2_)]SO_4_}.6H_2_O.

The asymmetric unit contains a [Mn(H_2_O)_2_(C_12_H_8_N_2_)]^2+^ cation, one sulfate anion and six lattice H_2_O molecules (Fig. 1). In the complex cations, the coordination geometry about the Mn atom is best considered as distorted octahedral, defined by four N atoms of two 1,10-phenanthroline (phen) ligands and two H_2_O molecules at the *cis* positions. The [Mn(H_2_O)_2_(C_12_H_8_N_2_)]^2+^ cation can be found in several previously reported complexes (Devereux *et al.*, 2000; Zheng *et al.*, 2003; Zhang *et al.*, 2003; Zhang *et al.*, 2005), with a similar coordination geometry. The Mn-N bond distances fall in the range 2.250 (4) to 2.318 (4) Å, and the Mn-O bond distances are 2.146 (3) and 2.177 (3) Å (Zheng *et al.*, 2003), respectively (Table 1). The *cisoid* and *transoid* angles about the central Mn atom vary from 74.06 (1) - 107.21 (2)° and 156.21 (2) - 166.50 (2)° (Table 1), respectively. All the bonding parameters are normal according to the similar coordination geometries reported. This fact indicates that the octahedral coordination of Mn atoms is severely distorted. Around the central Mn atom, both chelating phen planes orientate nearly perpendicularly to each other dihedral angle: 86.29 (8)°. The complex cations are arranged in such a way that each phen ligand containing N1 and N2 atoms are sandwiched by two symmetry-related, antiparallel phen ligands from different cations with the distances of 3.410 (6) Å forming a chain along the [100] direction, and along the [001] direction the phen ligand containing N3 and N4 atoms face to only one symmetry-related phen of the cation in next chain with the distance of 3.465 (5) Å. This implies that significant intermolecular π–π stacking interactions play vital roles in assembling the complex cations into two-dimensional positively charged layers parallel to (010) (Fig. 2). What's more, the sulfate anions and crystal water molecules form two-dimensional negatively charged layer parallel to (010) (Fig. 3) through extensive hydrogen bonds (Table 2).

As shown in Fig. 4, the the positive and negative two-dimensional sheets arrange alternatively and the two coordinational water molecules in the positive layers share their H atoms with O3 and O5 in sulfate anions and O7 of one lattic water molecule (Table 2) forming three-dimensional architecture. Hence, the crystal structure is further stabilized by interlayer hydrogen bonds. Both the positive and negative two-dimensional sheets are stack along the [010] direction in an ···ABAB··· sequence, and the layers A is shifted by a along the [100] direction with respect to the layers B (Fig. 4).

### Experimental

MnSO_4_.H_2_O (0.2253 g, 1.330 mmol), H_2_NCH_2_COOH (0.1009 g, 1.330 mmol) and 1,10-phenanthroline mono-hydrate (0.2644 g, 1.330 mmol) were completely dissolved in 20 ml mixed solvent of H_2_O and CH_3_OH (Vw:Ve = 1:1) under stirring. The resulting yellow solution was further stirred for 5 min forming yellowish precipitate. After the suspension was filtrated, the filtrate was allowed to stand at room temperature. The yellow transparent crystals were obtained 10 days later.

### Refinement

H atoms bonded to C atoms were palced in geometrically calculated position and were refined using a riding model, with *U*_iso_(H) = 1.2 *U*_eq_(C). H atoms attached to O atoms were found in a difference Fourier synthesis and were refined using a riding model, with the O–H distances fixed as initially found and with *U*_iso_(H) values set at 1.2 *U*eq(O).

### Crystal data

**Table:**

[Mn(C_12_H_8_N_2_)_2_(H_2_O)_2_]SO_4_·6H_2_O	*Z* = 2
*M**_r_* = 655.54	*F*(000) = 682
Triclinic, *P*1	*D*_x_ = 1.533 Mg m^−^^3^
Hall symbol: -P 1	Mo *K*α radiation, λ = 0.71073 Å
*a* = 10.153 (2) Å	Cell parameters from 13888 reflections
*b* = 12.086 (2) Å	θ = 3.0–27.5°
*c* = 13.309 (3) Å	µ = 0.61 mm^−^^1^
α = 109.55 (3)°	*T* = 293 K
β = 91.79 (3)°	Block, yellow
γ = 110.56 (3)°	0.29 × 0.24 × 0.19 mm
*V* = 1420.2 (5) Å^3^	

### Data collection

**Table:**

Rigaku R-AXIS RAPID diffractometer	6388 independent reflections
Radiation source: fine-focus sealed tube	5780 reflections with *I* > 2σ(*I*)
graphite	*R*_int_ = 0.022
Detector resolution: 0 pixels mm^-1^	θ_max_ = 27.5°, θ_min_ = 3.0°
ω scans	*h* = −13→13
Absorption correction: multi-scan (*ABSCOR*; Higashi, 1995)	*k* = −15→15
*T*_min_ = 0.680, *T*_max_ = 0.843	*l* = −17→17
13888 measured reflections	

### Refinement

**Table:**

Refinement on *F*^2^	Primary atom site location: structure-invariant direct methods
Least-squares matrix: full	Secondary atom site location: difference Fourier map
*R*[*F*^2^ > 2σ(*F*^2^)] = 0.048	Hydrogen site location: inferred from neighbouring sites
*wR*(*F*^2^) = 0.147	H-atom parameters constrained
*S* = 1.19	*w* = 1/[σ^2^(*F*_o_^2^) + (0.0566*P*)^2^ + 3.5944*P*] where *P* = (*F*_o_^2^ + 2*F*_c_^2^)/3
6388 reflections	(Δ/σ)_max_ = 0.001
382 parameters	Δρ_max_ = 0.56 e Å^−^^3^
0 restraints	Δρ_min_ = −0.58 e Å^−^^3^

### Special details

**Table:**

Geometry. All e.s.d.'s (except the e.s.d. in the dihedral angle between two l.s. planes) are estimated using the full covariance matrix. The cell e.s.d.'s are taken into account individually in the estimation of e.s.d.'s in distances, angles and torsion angles; correlations between e.s.d.'s in cell parameters are only used when they are defined by crystal symmetry. An approximate (isotropic) treatment of cell e.s.d.'s is used for estimating e.s.d.'s involving l.s. planes.
Refinement. Refinement of *F*^2^ against ALL reflections. The weighted *R*-factor *wR* and goodness of fit *S* are based on *F*^2^, conventional *R*-factors *R* are based on *F*, with *F* set to zero for negative *F*^2^. The threshold expression of *F*^2^ > σ(*F*^2^) is used only for calculating *R*-factors(gt) *etc*. and is not relevant to the choice of reflections for refinement. *R*-factors based on *F*^2^ are statistically about twice as large as those based on *F*, and *R*- factors based on ALL data will be even larger.

### Fractional atomic coordinates and isotropic or equivalent isotropic displacement parameters (Å^2^)

**Table:**

	*x*	*y*	*z*	*U*_iso_*/*U*_eq_	Occ. (<1)
Mn	0.74982 (5)	0.06218 (4)	0.25508 (3)	0.01016 (12)	
O1	0.7338 (2)	0.2453 (2)	0.33132 (17)	0.0170 (4)	
H1B	0.6970	0.2809	0.2994	0.020*	
H1C	0.7792	0.3034	0.3923	0.020*	
O2	0.5755 (2)	0.0179 (2)	0.13690 (17)	0.0160 (4)	
H2B	0.5868	0.0808	0.1178	0.019*	
H2C	0.5332	−0.0509	0.0822	0.019*	
N3	0.8118 (3)	−0.1022 (2)	0.15971 (19)	0.0121 (5)	
N4	0.9536 (3)	0.1485 (2)	0.1978 (2)	0.0123 (5)	
C13	0.7410 (3)	−0.2248 (3)	0.1397 (2)	0.0150 (6)	
H13A	0.6498	−0.2503	0.1569	0.018*	
C14	0.7967 (3)	−0.3184 (3)	0.0935 (2)	0.0174 (6)	
H14A	0.7435	−0.4034	0.0811	0.021*	
C15	0.9308 (3)	−0.2821 (3)	0.0672 (2)	0.0173 (6)	
H15A	0.9698	−0.3424	0.0371	0.021*	
C16	1.0095 (3)	−0.1523 (3)	0.0864 (2)	0.0147 (6)	
C17	1.1511 (3)	−0.1066 (3)	0.0616 (2)	0.0166 (6)	
H17A	1.1942	−0.1635	0.0311	0.020*	
C18	1.2219 (3)	0.0193 (3)	0.0826 (2)	0.0178 (6)	
H18A	1.3137	0.0474	0.0670	0.021*	
C19	1.1588 (3)	0.1097 (3)	0.1280 (2)	0.0144 (6)	
C20	1.2288 (3)	0.2411 (3)	0.1497 (2)	0.0175 (6)	
H20A	1.3209	0.2732	0.1356	0.021*	
C21	1.1596 (3)	0.3203 (3)	0.1916 (3)	0.0186 (6)	
H21A	1.2036	0.4066	0.2047	0.022*	
C22	1.0223 (3)	0.2718 (3)	0.2150 (2)	0.0149 (6)	
H22A	0.9770	0.3274	0.2437	0.018*	
C23	1.0203 (3)	0.0679 (3)	0.1537 (2)	0.0115 (5)	
C24	0.9445 (3)	−0.0657 (3)	0.1328 (2)	0.0112 (5)	
N1	0.6023 (3)	−0.0581 (2)	0.3357 (2)	0.0127 (5)	
N2	0.8784 (3)	0.1072 (2)	0.4174 (2)	0.0130 (5)	
C1	0.4671 (3)	−0.1373 (3)	0.2970 (3)	0.0165 (6)	
H1A	0.4259	−0.1441	0.2307	0.020*	
C2	0.3832 (3)	−0.2111 (3)	0.3509 (3)	0.0204 (6)	
H2A	0.2891	−0.2655	0.3208	0.024*	
C3	0.4425 (4)	−0.2017 (3)	0.4487 (3)	0.0193 (6)	
H3A	0.3889	−0.2502	0.4855	0.023*	
C4	0.5847 (3)	−0.1184 (3)	0.4934 (2)	0.0163 (6)	
C5	0.6523 (4)	−0.1002 (3)	0.5975 (3)	0.0205 (7)	
H5A	0.6034	−0.1484	0.6362	0.025*	
C6	0.7860 (4)	−0.0138 (3)	0.6393 (3)	0.0209 (7)	
H6A	0.8266	−0.0014	0.7077	0.025*	
C7	0.8677 (3)	0.0597 (3)	0.5812 (2)	0.0154 (6)	
C8	1.0075 (3)	0.1515 (3)	0.6232 (2)	0.0190 (6)	
H8A	1.0509	0.1678	0.6920	0.023*	
C9	1.0791 (3)	0.2168 (3)	0.5617 (3)	0.0186 (6)	
H9A	1.1716	0.2772	0.5880	0.022*	
C10	1.0105 (3)	0.1909 (3)	0.4585 (2)	0.0153 (6)	
H10A	1.0602	0.2346	0.4170	0.018*	
C11	0.8066 (3)	0.0417 (3)	0.4773 (2)	0.0124 (5)	
C12	0.6609 (3)	−0.0479 (3)	0.4332 (2)	0.0122 (5)	
S	0.63189 (8)	0.32046 (7)	0.10157 (6)	0.01227 (16)	
O3	0.5795 (2)	0.1805 (2)	0.04230 (18)	0.0182 (5)	
O4	0.5424 (2)	0.3715 (2)	0.05688 (18)	0.0192 (5)	
O5	0.6208 (3)	0.3446 (2)	0.21746 (18)	0.0213 (5)	
O6	0.7820 (2)	0.3794 (2)	0.09130 (18)	0.0196 (5)	
O7	0.8819 (2)	0.4348 (2)	0.53612 (18)	0.0210 (5)	
H7A	0.9164	0.4367	0.5964	0.025*	
H7B	0.8201	0.4683	0.5535	0.025*	
O8	1.0157 (3)	0.5572 (2)	0.25819 (19)	0.0246 (5)	
H8B	0.9358	0.5072	0.2170	0.029*	
H8C	1.0653	0.5711	0.2094	0.029*	
O11	0.8003 (3)	0.3984 (3)	−0.1132 (2)	0.0327 (6)	
H11A	0.7345	0.4124	−0.1264	0.039*	
H11B	0.7949	0.3809	−0.0561	0.039*	
O10	1.3026 (3)	0.4366 (3)	0.4498 (3)	0.0414 (7)	
H10C	1.3469	0.4538	0.5125	0.050*	
H10B	1.3551	0.4521	0.4037	0.050*	
O9	0.5397 (3)	0.5248 (3)	0.3499 (3)	0.0487 (9)	
H9B	0.5623	0.4683	0.3043	0.058*	
H9C	0.5554	0.5789	0.3190	0.058*	
O12A	0.4571 (6)	0.5666 (5)	0.1826 (4)	0.0202 (8)	0.50
O12B	0.4397 (6)	0.5565 (5)	0.1271 (4)	0.0202 (8)	0.50
H12A	0.4689	0.4981	0.1028	0.024*	
H12B	0.4618	0.5773	0.0720	0.024*	

### Atomic displacement parameters (Å^2^)

**Table:**

	*U*^11^	*U*^22^	*U*^33^	*U*^12^	*U*^13^	*U*^23^
Mn	0.0099 (2)	0.0115 (2)	0.0100 (2)	0.00457 (16)	0.00130 (15)	0.00454 (17)
O1	0.0218 (11)	0.0155 (10)	0.0132 (10)	0.0094 (9)	−0.0018 (8)	0.0028 (8)
O2	0.0177 (11)	0.0131 (10)	0.0150 (10)	0.0055 (8)	−0.0044 (8)	0.0039 (8)
N3	0.0123 (11)	0.0120 (11)	0.0107 (11)	0.0039 (9)	0.0007 (9)	0.0035 (9)
N4	0.0121 (11)	0.0124 (11)	0.0114 (11)	0.0043 (9)	0.0003 (9)	0.0038 (9)
C13	0.0130 (14)	0.0153 (14)	0.0148 (14)	0.0039 (11)	0.0007 (11)	0.0051 (12)
C14	0.0202 (15)	0.0123 (14)	0.0171 (14)	0.0051 (12)	−0.0022 (12)	0.0039 (12)
C15	0.0218 (15)	0.0186 (15)	0.0144 (14)	0.0131 (13)	0.0020 (12)	0.0041 (12)
C16	0.0149 (14)	0.0193 (14)	0.0100 (13)	0.0081 (12)	−0.0003 (11)	0.0042 (11)
C17	0.0167 (14)	0.0248 (16)	0.0143 (14)	0.0140 (13)	0.0032 (11)	0.0080 (12)
C18	0.0104 (13)	0.0289 (17)	0.0152 (14)	0.0084 (12)	0.0035 (11)	0.0084 (13)
C19	0.0126 (14)	0.0193 (14)	0.0109 (13)	0.0054 (12)	−0.0001 (11)	0.0059 (11)
C20	0.0131 (14)	0.0203 (15)	0.0154 (14)	0.0004 (12)	0.0025 (11)	0.0084 (12)
C21	0.0181 (15)	0.0168 (14)	0.0176 (15)	0.0011 (12)	0.0011 (12)	0.0082 (12)
C22	0.0179 (14)	0.0128 (13)	0.0130 (13)	0.0045 (11)	0.0009 (11)	0.0052 (11)
C23	0.0117 (13)	0.0147 (13)	0.0075 (12)	0.0053 (11)	0.0000 (10)	0.0031 (11)
C24	0.0110 (13)	0.0135 (13)	0.0066 (12)	0.0045 (11)	−0.0020 (10)	0.0011 (10)
N1	0.0136 (12)	0.0128 (11)	0.0113 (11)	0.0057 (10)	0.0026 (9)	0.0034 (9)
N2	0.0148 (12)	0.0116 (11)	0.0129 (11)	0.0059 (10)	0.0017 (9)	0.0040 (10)
C1	0.0144 (14)	0.0187 (15)	0.0146 (14)	0.0046 (12)	0.0028 (11)	0.0057 (12)
C2	0.0153 (15)	0.0183 (15)	0.0209 (15)	0.0014 (12)	0.0066 (12)	0.0041 (13)
C3	0.0207 (16)	0.0143 (14)	0.0199 (15)	0.0034 (12)	0.0088 (12)	0.0059 (12)
C4	0.0198 (15)	0.0159 (14)	0.0157 (14)	0.0086 (12)	0.0069 (12)	0.0067 (12)
C5	0.0278 (17)	0.0248 (16)	0.0161 (15)	0.0123 (14)	0.0088 (13)	0.0133 (13)
C6	0.0261 (17)	0.0300 (17)	0.0148 (14)	0.0149 (14)	0.0055 (13)	0.0138 (14)
C7	0.0185 (15)	0.0191 (14)	0.0124 (13)	0.0110 (12)	0.0029 (11)	0.0064 (12)
C8	0.0205 (15)	0.0238 (16)	0.0134 (14)	0.0112 (13)	−0.0028 (12)	0.0051 (13)
C9	0.0149 (14)	0.0182 (15)	0.0193 (15)	0.0064 (12)	−0.0039 (12)	0.0035 (12)
C10	0.0158 (14)	0.0146 (14)	0.0137 (14)	0.0059 (12)	0.0006 (11)	0.0033 (11)
C11	0.0153 (14)	0.0128 (13)	0.0107 (13)	0.0076 (11)	0.0014 (11)	0.0040 (11)
C12	0.0139 (13)	0.0126 (13)	0.0120 (13)	0.0070 (11)	0.0042 (11)	0.0045 (11)
S	0.0131 (3)	0.0125 (3)	0.0120 (3)	0.0064 (3)	0.0007 (3)	0.0041 (3)
O3	0.0199 (11)	0.0144 (10)	0.0170 (11)	0.0047 (9)	−0.0016 (9)	0.0043 (9)
O4	0.0209 (11)	0.0241 (12)	0.0194 (11)	0.0146 (10)	0.0030 (9)	0.0102 (10)
O5	0.0306 (13)	0.0261 (12)	0.0143 (11)	0.0182 (11)	0.0062 (9)	0.0080 (10)
O6	0.0126 (10)	0.0210 (11)	0.0210 (11)	0.0021 (9)	−0.0005 (9)	0.0076 (9)
O7	0.0192 (11)	0.0223 (12)	0.0174 (11)	0.0058 (9)	0.0032 (9)	0.0049 (9)
O8	0.0263 (13)	0.0234 (12)	0.0177 (11)	0.0059 (10)	−0.0017 (10)	0.0044 (10)
O11	0.0397 (16)	0.0380 (15)	0.0203 (12)	0.0122 (13)	0.0049 (11)	0.0135 (12)
O10	0.0307 (15)	0.0387 (16)	0.0402 (17)	0.0067 (13)	0.0160 (13)	0.0033 (14)
O9	0.0339 (16)	0.0243 (14)	0.077 (2)	0.0142 (12)	0.0268 (16)	0.0006 (15)
O12A	0.0231 (17)	0.0188 (15)	0.025 (2)	0.0113 (13)	0.011 (2)	0.012 (2)
O12B	0.0231 (17)	0.0188 (15)	0.025 (2)	0.0113 (13)	0.011 (2)	0.012 (2)

### Geometric parameters (Å, °)

**Table:**

Mn—O2	2.119 (2)	C1—C2	1.403 (4)
Mn—O1	2.171 (2)	C1—H1A	0.9300
Mn—N4	2.251 (3)	C2—C3	1.368 (5)
Mn—N1	2.264 (3)	C2—H2A	0.9300
Mn—N3	2.279 (3)	C3—C4	1.405 (5)
Mn—N2	2.282 (3)	C3—H3A	0.9300
O1—H1B	0.8549	C4—C12	1.413 (4)
O1—H1C	0.8553	C4—C5	1.439 (4)
O2—H2B	0.8511	C5—C6	1.345 (5)
O2—H2C	0.8548	C5—H5A	0.9300
N3—C13	1.326 (4)	C6—C7	1.434 (4)
N3—C24	1.362 (4)	C6—H6A	0.9300
N4—C22	1.337 (4)	C7—C8	1.410 (5)
N4—C23	1.363 (4)	C7—C11	1.413 (4)
C13—C14	1.409 (4)	C8—C9	1.374 (5)
C13—H13A	0.9300	C8—H8A	0.9300
C14—C15	1.372 (5)	C9—C10	1.402 (4)
C14—H14A	0.9300	C9—H9A	0.9300
C15—C16	1.415 (4)	C10—H10A	0.9300
C15—H15A	0.9300	C11—C12	1.449 (4)
C16—C24	1.411 (4)	S—O6	1.471 (2)
C16—C17	1.440 (4)	S—O4	1.471 (2)
C17—C18	1.358 (5)	S—O5	1.486 (2)
C17—H17A	0.9300	S—O3	1.488 (2)
C18—C19	1.430 (4)	O7—H7A	0.8553
C18—H18A	0.9300	O7—H7B	0.8584
C19—C23	1.413 (4)	O8—H8B	0.8548
C19—C20	1.413 (4)	O8—H8C	0.8587
C20—C21	1.364 (5)	O11—H11A	0.7729
C20—H20A	0.9300	O11—H11B	0.8533
C21—C22	1.399 (4)	O10—H10C	0.8590
C21—H21A	0.9300	O10—H10B	0.8503
C22—H22A	0.9300	O9—H9B	0.8535
C23—C24	1.445 (4)	O9—H9C	0.8577
N1—C1	1.330 (4)	O12A—H12A	1.1482
N1—C12	1.359 (4)	O12B—H12A	0.8306
N2—C10	1.324 (4)	O12B—H12B	0.8628
N2—C11	1.358 (4)		
			
O2—Mn—O1	86.91 (9)	C19—C23—C24	119.4 (3)
O2—Mn—N4	108.48 (9)	N3—C24—C16	122.8 (3)
O1—Mn—N4	92.43 (9)	N3—C24—C23	117.7 (3)
O2—Mn—N1	90.40 (9)	C16—C24—C23	119.5 (3)
O1—Mn—N1	102.38 (9)	C1—N1—C12	117.8 (3)
N4—Mn—N1	156.70 (9)	C1—N1—Mn	126.8 (2)
O2—Mn—N3	95.75 (9)	C12—N1—Mn	115.40 (19)
O1—Mn—N3	165.99 (9)	C10—N2—C11	118.4 (3)
N4—Mn—N3	73.66 (9)	C10—N2—Mn	127.0 (2)
N1—Mn—N3	91.38 (9)	C11—N2—Mn	114.51 (19)
O2—Mn—N2	160.56 (9)	N1—C1—C2	123.5 (3)
O1—Mn—N2	85.55 (9)	N1—C1—H1A	118.3
N4—Mn—N2	89.74 (9)	C2—C1—H1A	118.3
N1—Mn—N2	73.81 (10)	C3—C2—C1	118.8 (3)
N3—Mn—N2	95.89 (9)	C3—C2—H2A	120.6
Mn—O1—H1B	125.4	C1—C2—H2A	120.6
Mn—O1—H1C	127.7	C2—C3—C4	119.8 (3)
H1B—O1—H1C	105.3	C2—C3—H3A	120.1
Mn—O2—H2B	110.5	C4—C3—H3A	120.1
Mn—O2—H2C	128.3	C3—C4—C12	117.4 (3)
H2B—O2—H2C	108.8	C3—C4—C5	122.8 (3)
C13—N3—C24	118.0 (3)	C12—C4—C5	119.7 (3)
C13—N3—Mn	127.2 (2)	C6—C5—C4	120.4 (3)
C24—N3—Mn	114.30 (18)	C6—C5—H5A	119.8
C22—N4—C23	118.0 (3)	C4—C5—H5A	119.8
C22—N4—Mn	126.4 (2)	C5—C6—C7	121.9 (3)
C23—N4—Mn	115.07 (19)	C5—C6—H6A	119.0
N3—C13—C14	123.4 (3)	C7—C6—H6A	119.0
N3—C13—H13A	118.3	C8—C7—C11	117.4 (3)
C14—C13—H13A	118.3	C8—C7—C6	123.2 (3)
C15—C14—C13	118.9 (3)	C11—C7—C6	119.4 (3)
C15—C14—H14A	120.6	C9—C8—C7	119.5 (3)
C13—C14—H14A	120.6	C9—C8—H8A	120.3
C14—C15—C16	119.5 (3)	C7—C8—H8A	120.3
C14—C15—H15A	120.2	C8—C9—C10	119.0 (3)
C16—C15—H15A	120.2	C8—C9—H9A	120.5
C24—C16—C15	117.4 (3)	C10—C9—H9A	120.5
C24—C16—C17	119.7 (3)	N2—C10—C9	123.2 (3)
C15—C16—C17	122.9 (3)	N2—C10—H10A	118.4
C18—C17—C16	120.3 (3)	C9—C10—H10A	118.4
C18—C17—H17A	119.9	N2—C11—C7	122.5 (3)
C16—C17—H17A	119.9	N2—C11—C12	118.4 (3)
C17—C18—C19	121.7 (3)	C7—C11—C12	119.1 (3)
C17—C18—H18A	119.2	N1—C12—C4	122.7 (3)
C19—C18—H18A	119.2	N1—C12—C11	117.8 (3)
C23—C19—C20	117.5 (3)	C4—C12—C11	119.5 (3)
C23—C19—C18	119.4 (3)	O6—S—O4	110.88 (14)
C20—C19—C18	123.1 (3)	O6—S—O5	109.53 (14)
C21—C20—C19	119.1 (3)	O4—S—O5	109.66 (13)
C21—C20—H20A	120.4	O6—S—O3	109.29 (14)
C19—C20—H20A	120.4	O4—S—O3	109.25 (14)
C20—C21—C22	120.1 (3)	O5—S—O3	108.16 (14)
C20—C21—H21A	120.0	H7A—O7—H7B	103.2
C22—C21—H21A	120.0	H8B—O8—H8C	98.4
N4—C22—C21	122.6 (3)	H11A—O11—H11B	109.4
N4—C22—H22A	118.7	H10C—O10—H10B	115.7
C21—C22—H22A	118.7	H9B—O9—H9C	100.2
N4—C23—C19	122.7 (3)	H12A—O12B—H12B	88.5
N4—C23—C24	117.9 (3)		

### Hydrogen-bond geometry (Å, °)

**Table:**

*D*—H···*A*	*D*—H	H···*A*	*D*···*A*	*D*—H···*A*
O1—H1B···O5	0.86	1.82	2.670 (4)	174
O1—H1C···O7	0.86	1.99	2.843 (3)	178
O2—H2B···O3	0.85	1.83	2.656 (3)	164
O2—H2C···O3^i^	0.86	1.84	2.684 (3)	168
O7—H7A···O8^ii^	0.86	2.00	2.856 (3)	176
O7—H7B···O10^ii^	0.86	1.98	2.799 (4)	160
O8—H8B···O6	0.86	2.01	2.842 (4)	165
O8—H8C···O11^iii^	0.86	1.93	2.778 (4)	171
O9—H9B···O5	0.85	1.85	2.704 (4)	174
O9—H9C···O12A	0.86	1.98	2.617 (7)	131
O10—H10B···O9^iv^	0.85	2.04	2.836 (5)	157
O10—H10C···O9^ii^	0.86	2.02	2.875 (5)	172
O11—H11A···O12B^v^	0.77	1.93	2.691 (7)	166
O11—H11B···O6	0.85	1.98	2.813 (4)	167
O12A—H12A···O4	1.15	1.87	2.827 (6)	138
O12B—H12B···O4^v^	0.86	2.01	2.851 (6)	164

Symmetry codes:  (i) −*x*+1, −*y*, −*z*; (ii) −*x*+2, −*y*+1, −*z*+1; (iii) −*x*+2, −*y*+1, −*z*; (iv) *x*+1, *y*, *z*; (v) −*x*+1, −*y*+1, −*z*.
